# Respiration of Children in Reversed Presentations

**Published:** 1829

**Authors:** 


21. *Respiration of Children in Reversed Presentation.* By Professor Bigelow. Every obstetrical practitioner has experienced the anxieties excited in the closing stages of reversed presentations, by the perception that the fœtus is about to perish from the unavoidable compression of the funis after the body has been delivered. We have, ourselves, repeatedly felt this embarrassment, and more than once seen the young stranger perish, at the moment of his transition from a parasitic to an independent existence. Hence we have always regarded foot presentations and artificial footling cases, with great disrelish. Our practice in these critical circumstances, has been to introduce two or more fingers of the left hand into the posterior part of the os externum, with the back turned to the perinæum, and if possible carry them high enough to traverse the mouth of the child, when by forcibly pushing off the vagina, we may frequently open and maintain a passage to the ærial orifices, and establish the function of respiration. For obvious reasons, the umbilical cord should be brought and kept between the hand and the face of the fœtus. Our object, however, is not to detail the results of our own experience, but those of the learned and respectable professor, whose name we have quoted. It will be seen that they embrace the method which we have pursued, with other and more complicated measures. The following extended extract from his paper, in the August No. (1829) of the American Journal, sets forth all that he proposes:

The method to be pursued in conveying air to the mouth, depends upon the situation of the head. If the chin has descended low in the pelvis, so that the mouth rests upon the perinæum or lower part of the sacrum, and can be readily reached by the fingers, the hand of the operator alone is sufficient to give the assistance required. But if the mouth is situated so high in the pelvis as to be reached with difficulty, or if, from the large relative size of the head, there is much compression, the assistance of a tube may be of use. The mode of proceeding which I have found successful in various instances is as follows: as soon as the body and arms are extracted, supposing the face towards the sacrum, an assistant supports the body, carrying it towards the pubis; or the reverse, should the position of the face be to the pubis. The accoucheur should then introduce the hand to which the face looks, till the middle fingers rest upon the mouth of the child. The hand is then to be raised from the throat of the child, making the ends of the fingers a fulcrum, and pushing the perinæum backwards. The air will thus pass upwards as far as the chin of the child. The middle fingers are now to be separated about half an inch from each other, and thus a complete passage will be formed between them, by which the air will reach the mouth of the child. If the child be in a healthy state up to this period, it will immediately breathe and cry, and the delivery of the head may be safely postponed until the natural pains recur. If, from any degree of asphyxia, the child does not immediately breathe, it may often be made to do so by dashing cold water upon the body, or by other stimulating processes. It has even appeared to me practicable to inflate the lungs, in some cases, through an elastic catheter. When the mouth is so high in the pelvis as to be reached with difficulty, or when the compression is so great as to obliterate the cavity between the fingers, a flat tube will be found useful, made of metal, of spiral wire covered with leather, or of elastic gum, and having its largest diameter about half an inch. If the tube be of metal, or of any incompressible material, it should be withdrawn during a pain, to prevent contusion of the soft parts, and immediately replaced, if the pain subsides without expelling the head. Such a tube may be considered as a prolongation of the trachea, and is fully sufficient to sustain life by respiration for a considerable time. The tube must be guarded and directed by keeping it between the fingers of the inserted hand.—p. 286.

